# Impact of X-Ray Exposure From Computed Tomography on Wearable Insulin Delivery Devices

**DOI:** 10.1177/19322968231169722

**Published:** 2023-04-26

**Authors:** Frank Dong, Paul Johnson, Grant Fong, Alex Nguyen, Felipe Lauand, Todd Vienneau

**Affiliations:** 1 The University of Texas MD Anderson Cancer Center, Houston, TX, USA; 2 Imaging Institute, Cleveland Clinic, Cleveland, OH, USA; 3 Insulet Corporation, Acton, MA, USA

**Keywords:** computed tomography, radiation exposure, wearable insulin delivery system, functional integrity, X-ray

## Abstract

**Background:**

To investigate the impact of radiation exposure from a computed tomography (CT) scanner on the functional integrity of a wearable insulin delivery system.

**Methods:**

A total of 160 Omnipods and four personal diabetes managers (PDMs) were evenly divided into four groups: (1) control group (no radiation exposure), (2) typical radiation exposure group, (3) 4× typical radiation exposure group, and (4) scatter radiation group. Pods were attached to an anthropomorphic torso phantom on the abdomen (direct irradiation) or shoulder (scatter radiation) region. A third-generation dual-source CT scanner was used to scan the pods using either a typical exposure (used for routine CT abdominal study of a median size patient) or 4× typical exposure. A manufacturer-recommended 20-step functionality test was performed for all 160 Omnipods.

**Results:**

The radiation dose (measured in volume CT Dose index) was 16 mGy for a typical exposure, and 64 mGy for 4× typical exposure. The scatter radiation is less than 0.1 mGy. All Pods passed the functionality test except one pod in the scatter radiation group, which sounded an alarm due to occlusion. The blockage to the fluid was due to a kink in the soft cannula, a mechanical issue not caused by the radiation exposure.

**Conclusions:**

This study suggests X-ray exposure levels used in radiological imaging procedures do not negatively impact the functional integrity of Omnipods. This finding may support the potential for the manufacturer to remove the warning that patients should remove the Pod for X-ray imaging procedures, which will have a huge impact on patient care.

## Introduction

**Figure 1.**
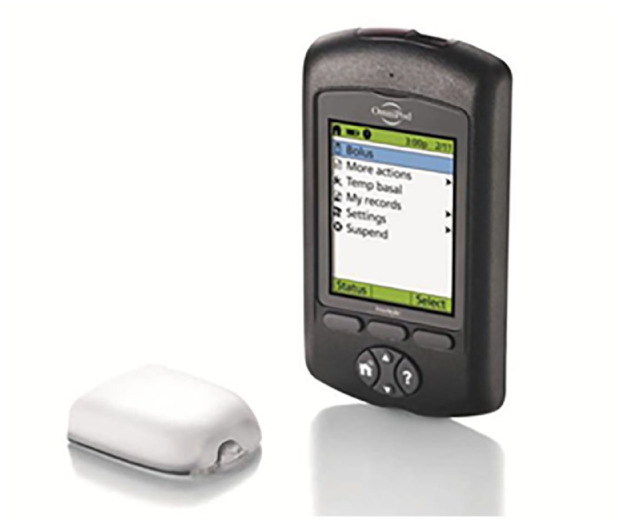
Omnipod pod and Omnipod personal diabetes manager (PDM).

Wearable insulin delivery devices such as the Omnipod Insulin Management System (Insulet Corp., Acton, MA) are popular among people living with diabetes. Omnipod is a tubeless, continuous insulin delivery device with two parts; a waterproof (IP28) wearable insulin patch pump (Pod) and a hand-held personal diabetes manager (PDM) (Figure 1). The PDM connects wirelessly to allow the user to initiate the Pod, change settings, and deliver boluses. Patients with a wearable insulin delivery system can perform physical activities such as swimming or jogging, while maintaining appropriate insulin delivery.

However, managing these wearable insulin delivery devices during radiological imaging procedures can be very challenging because manufacturer’s instructions for use generally require these devices be removed during these procedures.^1^ Once the device is removed, it cannot be re-used. This adds cost and inconvenience to patients as well as to the hospital because the imaging procedure may have to be re-scheduled if the patient is not willing to remove the device.

The concern about possible radiation interference or damage is mainly based on the assumption that X-ray photons may interact with semiconductor materials of micro-chips inside the wearable or implantable devices. As mentioned in the review article by Thomas and Heinemann,^2^ the extent of the interference or damage depends on how much energy per volume is deposited by the radiation. Compared to other forms of ionization radiation (such as alpha or beta particles) with similar initial energy, X-ray photon is more penetrating, that is, it deposits its energy along a much longer path length, and therefore, less energy per unit length. As chips become more miniature, the possibility of X-ray photons causing device malfunction may become higher, because the amount of deposited energy needed to interfere with these micro-chips becomes smaller. However, there are no data or evidence from the device manufacturers to prove that the radiation levels from the radiological imaging procedures are sufficient to cause interference with these devices, to justify the recommendation of removal during the radiological imaging procedures. These recommendations are mainly based on precautions when the data are lacking.

There have been several studies looking into the impact of radiological procedures on wearable or implantable devices.^3-7^ One study investigated the response of a variety of models of implantable cardiac rhythm management devices to the radiation delivered by computed tomography (CT).^3^ They found CT irradiation at typical clinical doses results in oversensing in most of the devices tested; however, the oversensing effects were predominantly transient and only detectable under direct X-ray irradiation. Another study investigated the safety and functional integrity of continuous glucose monitoring, Dexcom G6 (Dexcom, San Diego, CA) components after simulated radiologic procedures.^6^ The CGM devices were irradiated with a therapeutic beam of 6 MeV photons from a particle accelerator at the highest rate used in radiotherapy for an accumulative dose of 80 Gy. Compared to the devices in the control group, i.e., without radiation exposure, the difference of the mean glucose readings between the exposed group versus the control group is less than 1 mg/dL, and the coefficient of variance is much less than 1%. The results indicated that even under the radiation exposure level 5000× higher than those from a typical diagnostic CT procedure, the impact of radiation on CGM devices is negligible. In yet another study, a group from Japan researched the integrity of data recorded by flash glucose monitoring systems^7^ (FreeStyle Libre Pro, Abbott) following the exposure to chest X-ray, CT, radiation therapy (RT), and magnetic resonance imaging (MRI). They found there were no unread data or errors when the sensors were read. No change was observed before and after the examination for all tests.

The U.S. Food and Drug Administration (FDA) also weighs in on the impact of radiation from CT on the insulin pump and other electronic medical devices.^8^ Per FDA, “the probability of an adverse event being caused by exposing these devices to CT irradiation is extremely low, and it is greatly outweighed by the clinical benefit of a medically indicated CT examination.” The recommendation is to move the insulin pump (if feasible) so that it remains outside of the direct CT X-ray beam throughout the exam. If the insulin pump cannot be moved out of the direct X-ray field, FDA recommends to check with the patient to see if it can be turned off for a short period of time during the CT exam and turn it back on once the exam is complete. If the device cannot be moved or turned off, FDA recommends to adjust the scan range so the pump is outside the field of exposure or use the lowest exposure technique possible.

It will have a huge impact on patient care if these wearable devices do not need to be removed during the radiological procedures. Ultimately, the device manufacturers need to work with FDA to change the labeling of these devices to indicate radiation has very minimal or no negative impact on the functional integrity of these devices, and therefore, they are safe for the X-ray imaging procedures.

The goal of this article is to investigate the impact of radiation exposure from a CT scanner on the functional integrity of a wearable insulin delivery system. We irradiated the Omnipod Pods with typical and high (4× typical) levels of radiation from an abdomen/pelvis CT scan as well as from scatter radiation when the Pod was outside the scan range.

## Methods

**Figure 2.**
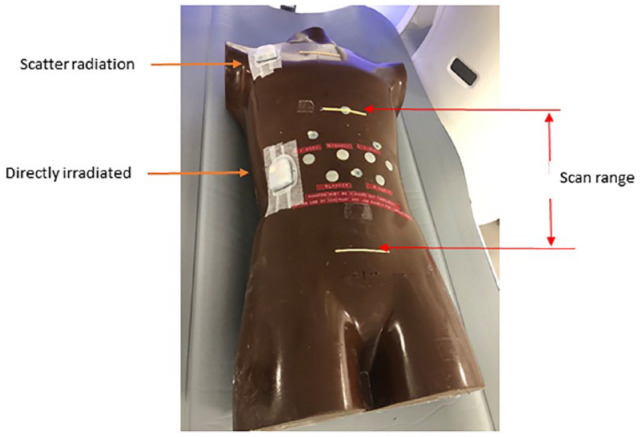
Pods were attached to the abdomen (for direct exposure) and shoulder (for scatter radiation) region. The CT scan range is identified by the upper (around diaphragm), and the lower (below the waist) strip which is radio-opaque (visible in a CT image). Abbreviation: CT, computed tomography.

An anthropomorphic torso phantom was used to simulate an average-sized patient; therefore, it is an *in vitro* study, and institutional review board (IRB) submission was not required. Per manufacturer’s testing instructions, saline was used as a surrogate for insulin during the testing. The Pods were attached to the abdomen and shoulder region (Figure 2) of the torso phantom during the CT scan. The Pod on the abdomen region was directly irradiated since it was in the mid-section of the scan range (between two radio-opaque yellow strips indicated with arrows in Figure 2). The Pod attached to the shoulder region was only exposed to the scatter radiation since it was outside the scan range.

One-hundred and sixty Pods were evenly divided into four groups: (1) a control group (not irradiated); (2) a typical exposure group (within a radiation field of a CT scan for an average patient size); (3) a high exposure group (4× typical exposure); (4) a scatter radiation group (outside the radiation field used for the high-exposure group).

A third-generation dual-source CT scanner Somatom Force (Siemens Healthineers, Erlangen, Germany) was used. For the typical exposure group, a routine abdominal scan protocol (Table 1) was used to scan the torso phantom. The scan range (between two parallel strips in Figure 2) was set so that the Pods were irradiated directly by the X-ray beam. For the high-exposure group, the scan technique was increased by 4× the tube current used in the typical exposure group with the same scan range and the location of the Pods as used in the typical exposure group. The Pods in the scatter radiation group were attached to the shoulder region outside the scan range. As the scatter radiation is in general proportional to the primary radiation, the Pods in this group were used in conjunction with the high-exposure group so maximum scatter radiation impact on the devices could be evaluated.

**Table 1.** Scan Acquisition Parameters for Typical, 4× Typical and Scatter Radiation Group.

Group	kVp	mA	Rotation time (s)	Pitch factor	Detector configuration
Typical exposure	120	240	0.5	0.6	96 × 0.6 mm
High (4× typical) exposure	120	960	0.5	0.6	96 × 0.6 mm
Scatter radiation	120	960	0.5	0.6	96 × 0.6 mm

The PDM was configured per manufacturer’s recommended settings. Before the CT scan, the Pods were filled with 200 units of saline, activated and paired with the PDM. During activation, the cannula was deployed into the air. The Pods were then attached to either the abdominal location for direct radiation exposure or the shoulder location for scatter radiation exposure (Figure 2). After CT scan, the remaining steps of the functionality test were performed on the Pods from each group, and comparison of the test results was made between the three “irradiated” groups and the control group. Once the functionality test was completed, an Insulet Binary Format file (.ibf) containing information related to each testing step was downloaded to a USB drive. The Pod was then deactivated and could not be re-used.

The functionality test case simulates actual use of the system by the patient. This test case verifies proper functionality of the features that patients will use most often on a daily basis. These features include communication functions: (1) activation, (2) deactivation, and (3) status update; and insulin delivery functions: (1) basal delivery; (2) bolus calculation and delivery; and (3) manual suspend and resume. For each Pod, the functionality test takes about 12 minutes to complete.

## Results

The CT scanner’s radiation output is measured by the volume CT dose index (CTDIvol), in the unit of mGy. For the typical exposure group, CTDIvol displayed on the scanner is 16 mGy, which is within the range of CTDIvol values of an abdominal CT study for an average-sized patient. The CTDIvol for a high-dose group was 64 mGy, which is considered high radiation exposure for an abdominal CT study, only used for very obese patients. The exposure to the Pods in the scatter radiation group is less than 0.1 mGy.

Results of the functionality testing are provided in Table 2. The Pods from the four groups (160 Pods in total) passed all functionality testing steps (20 steps in total) except for one Pod (lot number L72246, Serial number: 2080017) assigned to the scatter radiation group. In that case, the Pod passed the initial activation step. However, once the Pod was removed from the shoulder area after the scan, an alarm went off indicating the Pod should be deactivated. A hazard error message was also displayed in the PDM, and the functionality testing could not be completed.

**Table 2.** Results of the functional integrity testing.

	Functionality	Control group	Typical exposure (CTDIvol =16mGy)	High exposure (CTDIvol =64mGy)	Scatter radiation
Communication	Activation	All pass	All Pass	All Pass	All Pass
	Status update	All pass	All Pass	All Pass	Pass (except one pod alarmed)^a^
	Deactivation	All pass	All Pass	All Pass	Pass (except one pod alarmed)^a^
Insulin delivery	Basal	All pass	All Pass	All Pass	Pass (except one pod alarmed)^a^
	Bolus	All pass	All Pass	All Pass	Pass (except one pod alarmed)^a^
	Suspension/resume	All pass	All Pass	All Pass	Pass (except one pod alarmed)^a^

There were 40 Pods tested in each of the four groups, with a total of 160 Pods. Only one Pod in the scatter radiation group alarmed and the functionality testing could not be completed.

Abbreviation: CTDIvol, CT dose index.

a Alarm from the same pod.

The Pod that alarmed, as well as the ibf data file downloaded from the PDM was sent back to the manufacturer for investigation. The thorough inspection of the Pod by the engineer identified an occlusion, likely due to an observed kink in the soft cannula, which caused the fluid blockage. The alarm was sounded per design to alert patient of the blockage during insulin delivery. The occlusion was caused by a mechanical issue, not by the scatter radiation exposure from CT.

## Discussion

As glucose monitoring and insulin delivery systems continue to be miniaturized as wearable devices for the comfort of patients, they also bring several challenges during radiological imaging procedures. First, almost all manufacturers label these devices as contraindicated to radiation and require patients to remove these devices prior to the imaging procedures. Second, these wearable devices typically cannot be re-used once removed, therefore incurring financial burden to patients. Patients may also decide to postpone the imaging procedures, which may delay the diagnosis of diseases. Also, if the patient does remove the device for the procedure, a 30-min interruption of basal insulin infusion may result in significant glucose elevation with impact on the glucose control up to 3 hours after restarting delivery.^9^

This study has shown that the radiation exposure from a typical abdominal CT scan has no effect on the functional integrity of Omnipod Pods. Further testing using radiation exposure increased to 4× typical abdominal CT also revealed there was no effect to the functional integrity of Pods even under such high radiation field. The only Pod that alarmed happened in the group exposed by the scatter radiation, but the investigation by the engineer found there was an occlusion in the fluid delivery path due to a mechanical issue, not related to the radiation.

There are several limitations in this study. First, it tested only one manufacturer’s product. Different manufacturers may have different design of mechanical and electrical components used in wearable devices; therefore, future studies will show more varieties of such devices. The second limitation is that the testing only includes the functionalities used by patients, without looking into the details of any transient behavior of the electrical and mechanical components. Some abnormal activities may only exhibit during the duration of the radiation exposure. The third limitation, which is out of the scope of this investigation, is the effect of the radiation exposure on insulin related to its chemical and physical properties.

## Conclusions

This study suggests X-ray exposure levels used in radiological imaging procedures do not negatively impact the functional integrity of Omnipods. This finding may support the potential for the manufacturer to remove the warning that patients should remove the Pod for X-ray imaging procedures, which will have a huge impact on patient care.

## References

[1] Omnipod® Insulin Management System User Guide. Rev.004. Insulet Corporation; May 2021. https://www.omnipod.com/sites/default/files/2021-04/Omnipod-System_User-Guide_English.pdf.

[2] Thomas A Heinemann L . External physical and technical influences on medical devices for diabetes therapy [published online ahead of print February 22, 2022]. J Diabetes Sci Technol. doi:10.1177/19322968221080160. PMC10210111 35193431

[3] McCollough C Zhang J Primak A Clement WJ Buysman JR. Effects of CT irradiation on implantable cardiac rhythm management devices. Radiology. 2007;243(3):766-774. doi:10.1148/radiol.2433060993. 17463138

[bibr4-19322968231169722] Yamaji S Imai S Saito F Yagi H Kushiro T Uchiyama T. Does high-power computed tomography scanning equipment affect the operation of pacemakers? Circ J. 2006;70(2):190-197. doi:10.1253/circj.70.190. 16434814

[bibr5-19322968231169722] Mollerus M Naslund L Lipinski M Meyer A Libey B Dornfeld K. Radiation tolerance of contemporary implantable cardioverter-defibrillators. J Inter Card Electrophysiol. 2014;39(2):171-175. doi:10.1007/s10840-013-9861-z. 24317919

[bibr6-19322968231169722] Thomas C Welsh J Lu S Gray JM. Safety and functional integrity of continuous glucose monitoring components after simulated radiologic procedures. J Diabetes Sci Technol. 2021;15(4):781-785. doi:10.1177/1932296820920948. 32319318 PMC8252147

[7] Takatsu Y Shiozaki T Miyati T Asahara M Tani Y. Are the recorded data of flash glucose monitoring systems influenced by radiological examinations? Radiol Phys Technol. 2019;12(2):224-229. doi:10.1007/s12194-019-00505-x. 30811010

[8] U.S. Food and Drug Administration. Interference between CT and electronic medical devices. Silver Spring, MD; 2018. https://www.fda.gov/radiation-emitting-products/electromagnetic-compatibility-emc/interference-between-ct-and-electronic-medical-devices.

[9] Zisser H. Quantifying the impact of a short-interval interruption of insulin-pump infusion sets on glycemic excursions. Diabetes Care. 2008;31(2):238-239. doi:10.2337/dc07-1757. 18056889

